# STCCA: Spatial–Temporal Coupled Cross-Attention Through Hierarchical Network for EEG-Based Speech Recognition

**DOI:** 10.3390/s25216541

**Published:** 2025-10-23

**Authors:** Liang Dong, Hengyi Shao, Lin Zhang, Lei Li

**Affiliations:** 1 School of Artificial Intelligence, Beijing University of Posts and Telecommunications, Beijing 100876, China; 2 Beijing BigData Center, Beijing 101100, China

**Keywords:** cross-attention fusion, deep learning, EEG-based speech recognition, multi-head self-attention

## Abstract

Speech recognition based on Electroencephalogram (EEG) has attracted considerable attention due to its potential in communication and rehabilitation. Existing methods typically process spatial and temporal features with sequential, parallel, or constrained feature fusion strategies. However, the intricate cross-relationships between spatial and temporal features remain underexplored. To address these limitations, we propose a spatial–temporal coupled cross-attention mechanism through a hierarchical network, named STCCA. The proposed STCCA consists of three key components: local feature extraction module (LFEM), coupled cross-attention (CCA) fusion module, and global feature extraction module (GFEM). The LFEM employs CNNs to extract local temporal and spatial features, while the CCA fusion module leverages a dual-directional attention mechanism to establish deep interactions between temporal and spatial features. The GFEM uses multi-head self-attention layers to model long-range dependencies and extract global features comprehensively. STCCA is validated on three EEG-based speech datasets, achieving accuracies of 45.45%, 25.91%, and 29.07%, corresponding to improvements of 1.95%, 3.98%, and 1.98% over the comparison models.

## 1. Introduction

Language impairment caused by neurological damage is a severe disability that limits both work and social life [1,2]. The recognition of speech neural signals based on Electroencephalogram (EEG) offers an efficient communication and rehabilitation method for patients with language impairments [3,4,5]. As a widely used non-invasive technique for monitoring brain activity, EEG is characterized by its high temporal resolution and rich spatial–temporal features [6,7,8]. EEG-based speech recognition, encompassing inner speech, silent speech, and imagined speech recognition [9,10], holds significant potential for language impairments by decoding the intricate cross-relationships between spatial–temporal features [11,12].

Recently, various EEG-based speech recognition methods have been proposed, with deep learning (DL) methods taking a leading role, starting with convolutional neural networks (CNNs) [13,14]. CNNs have demonstrated exceptional capability in extracting local features [15], leading to their widespread adoption in EEG analysis. One of the most representative architectures is EEGNet [16,17], which proposes multiple convolution layer architectures to sequentially extract temporal and spatial representations from EEG signals. EEGNet has demonstrated notable performance across multiple EEG decoding tasks and remains a widely adopted baseline in the field. In addition to EEGNet, other representative models have emerged to enhance spatial–temporal representation learning. For instance, TSception [18] applies multi-scale temporal convolutions to capture temporal features, followed by asymmetric spatial convolutions to extract spatial representations. Despite these advancements, existing methods often treat parallel temporal and spatial features as independent entities, neglecting their inherent interconnections. To establish joint spatial–temporal relationships, several studies, including those by Chang et al. [19] and Chen et al. [20], have typically employed CNNs, dynamic connectogram to extract spatial and temporal features separately, and combined the parallel strategies such as feature summation, concatenation, or weighted averaging to fuse spatial–temporal features. In practice, the spatial locations of electrodes and the temporal evolution of EEG signals are tightly coupled due to the dynamic nature of cognitive and language-related brain activity. These universal approaches are inherently limited in capturing the intricate interactions and deep dependencies between spatial and temporal dimensions. In summary, existing approaches remain challenging due to the prevalent adoption of sequential, parallel, or constrained feature fusion strategies for processing temporal and spatial characteristics. Moreover, CNNs inherently operate with fixed-size receptive fields, which restrict their capacity to capture global context and long-range dependencies in EEG signals [21,22,23].

To address the aforementioned challenges, we propose a spatial–temporal coupled cross-attention mechanism within a hierarchical network, named STCCA, which is designed to extract and fuse local spatial–temporal features, and subsequently model long-range dependencies. The framework consists of three key components. First, the local feature extraction module (LFEM) leverages temporal and spatial convolutional layers to independently extract fine-grained temporal and spatial features from EEG signals, enabling effective representation of both temporal dynamics and spatial distributions. Second, the extracted features are fed into the coupled cross-attention (CCA) fusion module, where a bidirectional attention mechanism is employed to establish intricate joint relationships between temporal and spatial dimensions. The CCA facilitates the capture of joint spatial–temporal dependencies, addressing the limitations of traditional feature fusion approaches. Finally, the fused features are passed to the global feature extraction module (GFEM), which utilizes multi-head self-attention layers to model long-range dependencies, facilitating the representation of global spatial–temporal patterns. The hierarchically processed features are passed to the fully connected layers to output the recognition results. The main contributions of this letter can be summarized as follows:

1. We propose a novel CCA module, which applies attention mechanisms bidirectionally to two input features. To the best of our knowledge, this is the first CCA fusion method designed to fuse temporal and spatial features in EEG-based speech recognition.
2. We propose an innovative STCCA network that hierarchically captures local and global characteristics of EEG signals. The LFEM is employed to extract local temporal and spatial features, the CCA module fuses these features by capturing their coupled interactions, and the GFEM extracts global dependencies.
3. STCCA achieves accuracy improvements of 1.95%, 3.98%, and 1.98% on three EEG-based speech datasets with 22 subjects. Ablation experiments further validate the superior performance of the CCA compared to other commonly used fusion methods, and highlight the essential roles of LFEM and GFEM in enhancing overall performance.

The remainder of this article is organized as follows. Section 2 describes the proposed method, including LFEM, CCA, and GFEM. Section 3 presents the dataset preprocess, experiment settings, results and analyses, and ablation studies. Finally, Section 4 presents the conclusion and future work directions.

## 2. Method

### 2.1. Problem Definition

The research goal of EEG-based speech recognition is to train a brain-to-speech decoding network f:x→p, where the output $p\in R^{m}$ represents the coded representation of EEG signal across *m* target speech categories.

A set of EEG signal acquisition nodes $V=\left\{v_{1},v_{2},\ldots ,v_{ch}\right\}$ is spatially distributed over the scalp, where $ch=\left|V\right|$ denotes the number of electrode channels. Each node samples EEG signals at a frequency fs. The collected EEG signals are represented as $x\in R^{ch\times t}$, where *t* denotes the number of sampling time points. Each EEG segment is annotated with a ground-truth label $y\in \left\{1,2,\ldots ,m\right\}$, corresponding to a specific speech class. Fianlly, the extracted features are calculated into logits, resulting in predictions $\hat{y}\;\in \left\{1,2,\ldots ,m\right\}$.

### 2.2. Overview of STCCA

**Figure 1:**
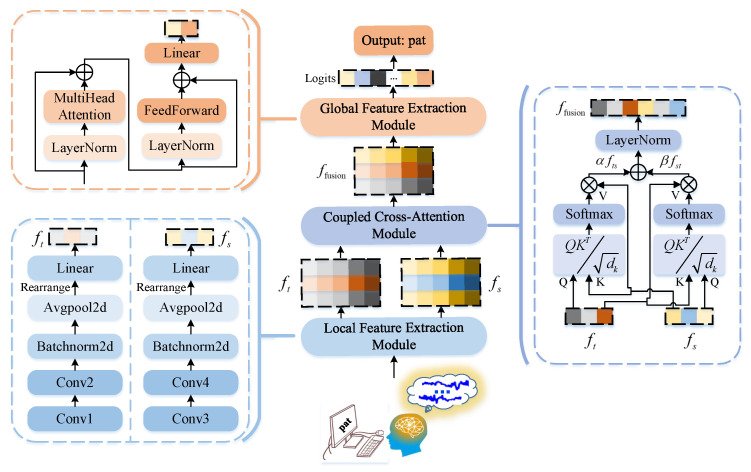
Architecture of the proposed STCCA.

The overall architecture of the proposed STCCA framework is illustrated in Figure 1, and its complete processing pipeline is outlined in Algorithm 1 using pseudo-code. The hierarchical design aims to first extract and fuse local spatial–temporal features, followed by modeling long-range dependencies for enhanced representation. The proposed method consists of three key components:

(1) LFEM. LFEM extracts local fine-grained temporal and spatial features from EEG signals through convolutional layers along the time and channel dimensions, enabling effective representation of both temporal dynamics and spatial distributions.
(2) CCA. CCA leverages a bidirectional attention mechanism to dynamically assign weights, enabling the modeling of complex interactions between temporal and spatial features. By capturing joint spatial–temporal dependencies, CCA effectively overcomes the limitations of traditional feature fusion approaches.
(3) GFEM. GFEM employs multi-head self-attention layers to model long-range dependencies, thereby enhancing the representation of global spatial–temporal patterns. The final classification results are then obtained through the fully connected layers.

**Algorithm 1:** Pseudo-code of STCCA
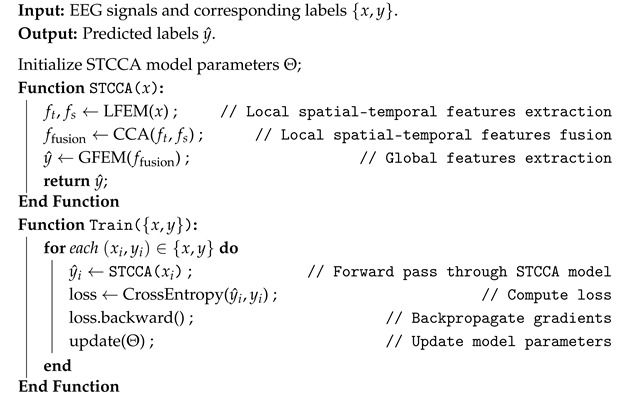


### 2.3. Local Feature Extraction Module

In view of the fact that neural activity is continuous, extracting local features from EEG signals plays a crucial role in improving EEG decoding performance. In the LFEM, two separate convolutional pathways are designed to capture both temporal and spatial local characteristics of the input EEG signals, as shown in Figure 1. Each convolutional pathway consists of convolutional layers (Conv), average pooling layers (AG), batch normalization layers (BN), and ELU activation functions. In the temporal convolutional pathway, temporal–spatial joint features ($f_{t}$) are calculated by Equation (1) along the time and channel dimensions. Similarly, in the spatial convolutional pathway, spatial–temporal joint features ($f_{s}$) are extracted based on Equation (2) along the channel and time dimensions. The parameters of LFEM are shown in Table 1.

(1) $$f_{t}=\mathrm{AG}\left(\phi \left(\mathrm{BN}\left(\mathrm{Conv}2\left(\mathrm{Conv}1(x)\right)\right)\right)\right),$$

(2) $$f_{s}=\mathrm{AG}\left(\phi \left(\mathrm{BN}\left(\mathrm{Conv}4\left(\mathrm{Conv}3(x)\right)\right)\right)\right),$$

where *x* is the input EEG signal, and ϕ is the activation function (ELU).

### 2.4. Coupled Cross-Attention Fusion Module

The $f_{t}$ and $f_{s}$ obtained from the LFEM are fed into the CCA fusion module. This module utilizes a bidirectional attention mechanism to explore the intrinsic relationships between the temporal and spatial dimensions, dynamically allocating weights to the spatial–temporal features, thereby achieving a comprehensive fusion of spatial–temporal complementary features. Specifically, CCA enables temporal query to focus on spatial patterns and spatial query to capture temporal information, enhancing the learning of context-aware interactions between spatial and temporal representations. The entire process is illustrated in Figure 1.

In the first direction of attention, the $f_{s}$ serves as the key and value, respectively, and the $f_{t}$ is designated as the query. The basic Scaled Dot-Product Attention [24] is applied, defined as

(3) $$\mathrm{Atten}\left(Q,K,V\right)=\mathrm{softmax}\left(\frac{QK^{T}}{\sqrt{d_{k}}}\right)V,$$

where *Q*, *K*, and *V* represent the query, key, and value, respectively, each with a shape of $R^{N_{b}\times 512\times k}$. Here, $N_{b}$ is the batch size, 512 represents the sequence length, and *k* is the feature dimension after linear projection, which is equal to the output dimension of the convolutional layer in Table 1. $\sqrt{d_{k}}$ is the size of the query. In this direction, temporal features $f_{t}$ act as query to focus on spatial patterns $f_{s}$, enabling the model to selectively emphasize spatial components that are most relevant at each time step. The formula is defined as follows:

(4) $$f_{ts}=\mathrm{Atten}\left(f_{t},f_{s},f_{s}\right),$$

In the second direction of attention, $f_{s}$ acts as the query, while $f_{t}$ is used as both the key and value, and the Equation (3) operation applies. This reverse attention allows spatial features to capture the temporal dynamics that is most informative for each spatial patterns, facilitating better temporal–contextual representation learning. The formula is defined as follows:

(5) $$f_{st}=\mathrm{Atten}\left(f_{s},f_{t},f_{t}\right)),$$

Finally, the $f_{ts}$, $f_{st}$ results from both directions are aggregated to produce the fused feature with learnable weight parameters, and layer normalization (LN) is applied to normalize the fused feature. The formula is defined as follows:

(6) $$f_{\mathrm{fusion}}=\mathrm{LN}\left(\alpha f_{ts}+\beta f_{st}\right),$$

where α and β are learnable unconstrained scalar weights for $f_{ts}$ and $f_{st}$, respectively.

### 2.5. Global Feature Extraction Module

Due to the fixed receptive field size, convolutional layers can only capture local temporal and spatial features, limiting their ability to extract global features from EEG signals. In the GFEM, we use multi-head self-attention (MHA) mechanism to learn global dependencies of EEG features, complementing the limited receptive field in the LFEM, as shown in Figure 1. The $f_{\mathrm{fusion}}$ are divided into *h* segments. Each segment is linearly transformed into global query (GQ), global key (GK), and global value (GV). The linear projection dimension is *k*. This process can be formulated as

(7) $$\begin{array}{cc} f_{g} & =\mathrm{MHA}(GQ,GK,GV)\\ \; & =[head_{0},\ldots ,head_{h-1}],\\ head_{l} & =\mathrm{Atten}(GQ_{l},GK_{l},GV_{l}), \end{array}$$

where $GQ_{l},GK_{l},GV_{l}$ denote different GQ,GK,GV in the $l_{th}$ head, respectively. The $GQ_{l},GK_{l},GV_{l}$ are processed by Equation (3), and all head outputs are concatenated.

(8) $$\hat{y}=\mathrm{argmax}\left(\mathrm{FC}\left(f_{g}\right)\right),$$

where $\hat{y}$ is the predicted labels.

## 3. Experiment and Results

### 3.1. EEG Data and Preprocessing

In this study, three datasets are applied to evaluate the effectiveness of the proposed STCCA. The key characteristics of these datasets are summarized in Table 2. Appendix A.1 reports the number of trials per class per subject for each dataset to illustrate the data distribution in detail. A detailed description of each dataset is presented in the following paragraphs.

Dataset I: Nieto et al. [9] developed an inner speech EEG dataset consisting of 10 native Spanish-speaking participants. EEG signals were collected using the 10–20 system with 128 EEG channels at a sampling rate of 1024 Hz. Participants were instructed to silently imagine four Spanish words: “arriba” (up), “abajo” (down), “derecha” (right), and “izquierda” (left). During preprocessing, the data were re-referenced by earlobe channels and subjected to band-pass filtering (0.5–100 Hz), along with a 50 Hz notch filter. The sampling rate was downsampled to 256 Hz. Independent Component Analysis (ICA) was applied to eliminate artifacts and ensure the integrity of the signal.

Dataset II: Zhao et al. [25] collected EEG signals, facial expressions, and voice patterns while subjects silently imagined four words (“pat,” “pot,” “knew,” and “gnaw”) and seven syllables (“iy,” “uw,” “piy,” “tiy,” “diy,” “m,” and “n”), forming the Kara One dataset. The EEG signals were recorded at a sampling frequency of 1000 Hz using the 10–20 system with 62 EEG channels. In this study, EEG data from six subjects were randomly selected for analysis. No further filtering or artifact correction was applied to the publicly available dataset provided by the authors.

**Figure 2:**
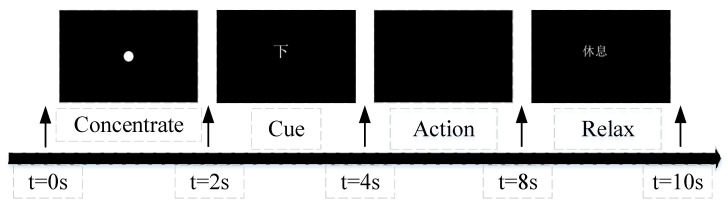
Trial workflow in each session. Chinese words “我”, “休息” correspond to “down”, “relax”, respectively.

Dataset III: To complement the publicly available dataset, we collected a private inner speech EEG dataset with 32 EEG channels at a sampling rate of 1000 Hz. Subjects were instructed to silently imagine the ten most frequently used Chinese words, which were “我” (I), “你” (you), “他” (he), “上” (up), “下” (down), “左” (left), “右” (right), “是” (are), “水” (water), and “饭” (food). Each subject completed five sessions, with each session comprising 100 trials. As illustrated in Figure 2, each trial consists of a 2-s concentrate period, a 2-s Chinese words cue presentation, a 4-s silent action imagination period, and a final 2-s relaxation period. During preprocessing, the data were re-referenced using CPz channel and filtered with a band-pass filter ranging from 0.5 to 100 Hz, along with a notch filter at 50 Hz. The sampling rate was then downsampled to 250 Hz. ICA was employed to remove artifacts, ensuring signal quality. This study complied with the Declaration of Helsinki and was conducted according to the guidelines of the Declaration of Helsinki and approved by the Ethics Committee of the Beijing University of Posts and Telecommunications (Ethic approval code: 202302003).

### 3.2. Experimental Setting

**Figure 3:**
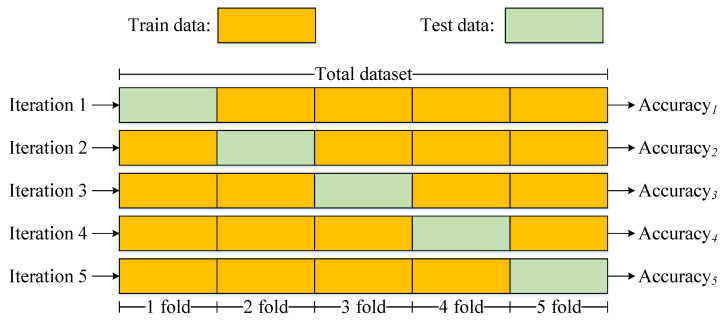
Schematic diagram of five-fold cross-validation.

The three datasets, consisting of Dataset I with four classes, Dataset II with eleven classes, and Dataset III with ten classes, are evaluated using five-fold cross-validation [26]. Specifically, all EEG trials of a subject are pooled and then randomly divided into five folds. The training-to-testing ratio in each iteration is 4:1, as shown in Figure 3. To ensure class balance within each fold, StratifiedKFold is applied. Accuracy is used as the evaluation metric, and the final accuracy is expressed as the mean (μ) ± standard deviation (σ), calculated as follows:

(9) Accuracy=μ±σ

(10) $$\mu =\frac{1}{5}\sum_{i=1}^{5}{\mathrm{Accuracy}}_{i}$$

(11) $$\sigma =\sqrt{\frac{1}{5}\sum_{i=1}^{5}{\left({\mathrm{Accuracy}}_{i}-\mu \right)}^{2}}$$

where ${\mathrm{Accuracy}}_{i}$ denotes the accuracy obtained in the ith iteration. The Wilcoxon Signed Rank Test is applyed to analyze final accuracy statistical significance.

The implementation uses the PyTorch library (1.8.1) and runs in parallel on six NVIDIA 1080Ti GPUs. The input 2D data (channels × time samples) are standardized using Z-score normalization. Both ICA and Z-score normalization are estimated exclusively on the training data and then applied to the test data to avoid data leakage. In the proposed model, the output dimension of the convolutional layer is set to k=32, and the number of attention heads is set to h=4, which provides a balance between model complexity and representation capacity. The model is optimized using the Adam optimizer for 200 epochs, with a StepLR scheduler that reduces the learning rate by a factor of 0.5 every 50 epochs. A dropout rate of 0.3 is applied during the training process to prevent overfitting, and cross-entropy loss function is applied, defined as

(12) $$L=-\frac{1}{N_{b}}\sum_{i=1}^{N_{b}}\sum_{c=1}^{m}y\log\left(\hat{y}\right),$$

where *m* is the number of EEG categories, *y* and $\hat{y}$ are the ground-truth and predicted labels, and $N_{b}$ is the batch size.

### 3.3. Experimental Results and Analyses

**Figure 4:**
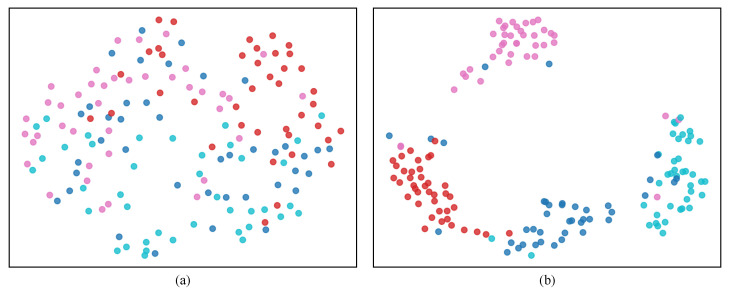
T-SNE visualizations of feature distributions at different training epochs on Dataset I: (**a**) 3 epochs; (**b**) 50 epochs. Different colors represent different feature categories.

**Figure 5:**
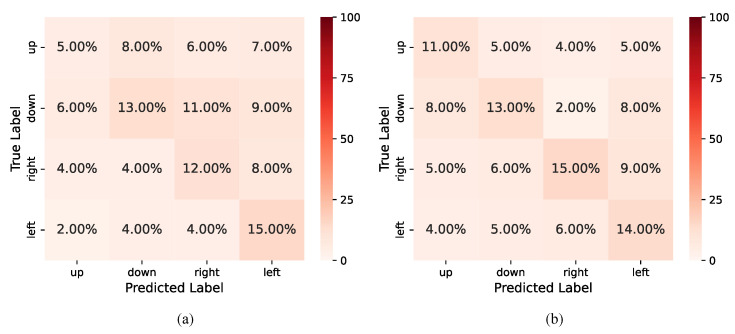
Confusion matrices of (**a**) S09 and (**b**) S10 on Dataset I.

To evaluate the effectiveness of STCCA, we conducted extensive experiments on three benchmark datasets. The performance of STCCA was compared with ten baseline methods: ShallowConvNet [27,28], DeepConvNet [27,28], EEGNet [17,29], RACNN [30], EEG-ChannelNet [31], Conformer [22], LMDA-Net [32], AISR(SPWVD+CNN) [33], EEG-Deformer [21], and D-FaST [20]. As illustrated in Figure 4, the feature distributions become increasingly compact and separable with the progression of training epochs. The test results for each dataset, summarized in Table 3, Table 4 and Table 5, demonstrate the superior performance of the proposed STCCA model compared to the baseline methods. Our proposed STCCA achieves the highest average accuracies of 45.45±6.48%, 25.91±4.84%, and 29.07±5.44% on Dataset I, Dataset II, and Dataset III. The results demonstrate that STCCA significantly outperforms the baseline methods, with accuracy improvements of 1.95% (*p* < 0.05), 3.98% (*p* < 0.05), and 1.98% (*p* < 0.05), respectively. These baseline methods can be broadly categorized into CNN-based and attention-based methods. CNN-based methods excel at capturing local spatial or temporal patterns, whereas attention-based methods are more effective at modeling global dependencies. However, these methods generally fail to explicitly capture the coupling relationships between temporal and spatial features. For example, methods such as EEGNet treat temporal and spatial features independently, extracting them sequentially without considering their mutual interactions. Similarly, methods like D-Fast employ constrained fusion strategies—such as direct concatenation of features—that are limited in their ability to capture the intrinsic interactions and deep dependencies between temporal and spatial features. In contrast, STCCA integrates the complementary strengths of both CNNs and attention mechanisms within a hierarchical network. It not only extracts local and global features effectively but also models the complex and complementary relationships between temporal and spatial features. As a result, STCCA achieves the highest average accuracy across all datasets (*p* < 0.05). Figure 5 presents the confusion matrix for the classification of two randomly selected subjects. The confusion matrices of all ten subjects are provided in Appendix A.2. The diagonal elements represent the probabilities of accurate classification for each respective category. These results demonstrate that STCCA achieves superior recognition performance compared to other models in three EEG-based speech recognition for 18 subjects on three datasets.

Furthermore, the F1_scores of STCCA are computed, as shown in Table 6, demonstrating stable classification performance across subjects. To further evaluate the sensitivity of STCCA, we conduct channel dropout experiments, where 5% and 10% of EEG channels are randomly removed. The results show a decline in accuracy under channel loss conditions, which can be attributed to the spatial–temporal fusion mechanism of STCCA, where dropping channels inevitably leads to the loss of spatial information. In addition, Table 7 compares the parameters, FLOPs, and inference time among several representative baseline models. Although STCCA introduces a moderate number of parameters (7.155 M) and computational cost (2581.882 MFLOPs), it achieves the fastest inference time (4.597 ms). Collectively, these results demonstrate that STCCA maintains competitive performance while remaining computationally efficient, making it suitable for EEG-based speech recognition.

To further evaluate the cross-subject generalization ability of STCCA, we conduct a Leave-One-Subject-Out (LOSO) cross-validation, in which one subject is used as the test set while the remaining subjects are used for training. As shown in Table 8, the proposed STCCA consistently achieves the highest or near-highest accuracies across most subjects, with an overall improvement over the baselines. Specifically, STCCA attains the best performance for seven out of ten subjects (S01, S02, S04, S05, S06, S08, and S10), indicating its strong generalization capability when tested on unseen subjects. Compared with classical CNN-based architectures (ShallowConvNet, DeepConvNet, and EEGNet) and attention-based models (Deformer and D-FaST), STCCA exhibits more stable accuracy across subjects, demonstrating its ability to effectively capture invariant spatial–temporal representations from heterogeneous EEG patterns. These results validate that the proposed model maintains reliable performance under cross-subject conditions in EEG-based speech decoding.

### 3.4. Ablation Studies

**Figure 6:**
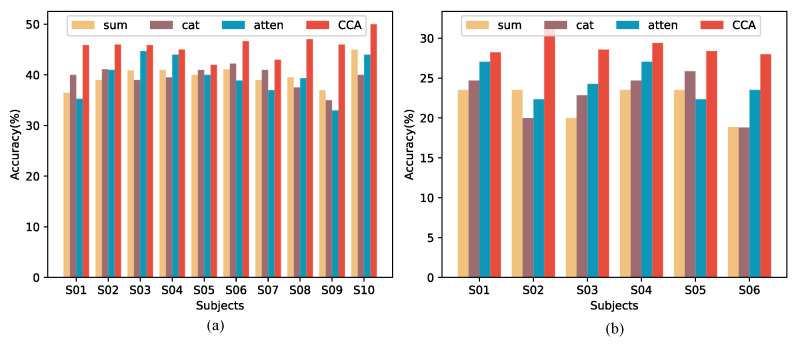
Contrast results of ablation experiments on (**a**) Dataset I and (**b**) Dataset III.

We conduct ablation experiments to evaluate the contribution of each module in STCCA. As shown in Table 9, Table 10 and Table 11, removing either LFEM or GFEM results in a notable decline in performance, highlighting the critical contribution of both modules to the hierarchical architecture. To further validate the contribution of the CCA module, we replace it with three commonly used feature fusion strategies: summation (sum), concatenation (cat), and standard attention (atten). Standard attention computes a weighted combination of the temporal and spatial features by applying a linear projection, followed by softmax normalization to obtain attention weights that are then used to produce the fused feature. These commonly used feature fusion strategies treat temporal and spatial features independently, or fuse them statically. Each modified model is evaluated on the same datasets under identical experimental conditions, and the results are shown in Figure 6. The proposed CCA mechanism demonstrates superior performance, achieving the highest average accuracy (*p* < 0.05) across both datasets. In contrast, the sum, cat, and atten strategies yield lower accuracies, indicating their limited ability to capture the intricate relationships between temporal and spatial features. This highlights the distinct advantage of CCA in modeling bidirectional interactions between temporal and spatial features, thereby enhancing the spatial–temporal joint features. In addition to performance, we compare computational cost and inference efficiency, as shown in Table 12. All strategies have the same number of parameters, and while CCA has slightly higher FLOPs, its inference time remains comparable, indicating that the accuracy gain comes with minimal computational overhead. These results confirm the critical role of CCA in STCCA, emphasizing its role in driving performance improvements in EEG-based speech recognition.

## 4. Conclusions

In this study, we propose the STCCA network, a novel hierarchical framework designed to tackle the challenges of spatial–temporal feature fusion and local-global dependency modeling for EEG-based speech recognition. By integrating the CCA mechanism, the STCCA effectively captures the intricate bidirectional relationships between spatial and temporal features. Furthermore, the combination of convolutional layers and self-attention enables the network to extract both local and global features hierarchically, leading to a comprehensive feature representation. However, the current implementation is limited to offline analysis and has not yet been extended to real-time online systems, which restricts its applicability in practical scenarios. In addition, as the dataset scale increases, the model may face challenges in convergence due to increased data complexity, potentially requiring longer training and more careful parameter tuning to maintain stability and performance. Future research will aim to optimize the framework for real-time processing and deployment, while incorporating adaptive optimization strategies to improve convergence efficiency on larger EEG datasets, facilitating its integration into practical applications. This study highlights the potential of STCCA as a powerful tool for advancing EEG signal decoding in speech recognition, thus paving the way for more effective brain–computer interface applications.

## Figures and Tables

**Table 1:** Parameters of the LFEM.

Layer Name	Layer Function	In	Out	Kernel	Stride
Conv1	Conv2d	1	k	(1, fs/10)	(1, 1)
Conv2	Conv2d	k	k	(ch/2, 1)	(1, 1)
Conv3	Conv2d	1	k	(ch/2, 1)	(1, 1)
Conv4	Conv2d	k	k	(1, fs/10)	(1, 1)
BN	Batchnorm2d	-	-	k	-
AG	Avgpool2d	-	-	(4, 4)	-

k is the output of the convolutional layer, and fs and ch mean the frequency and the electrode channels of EEG.

**Table 2:** The information about three datasets.

	Dataset I	Dataset II	Dataset III
Number of subjects	10	6	6
Number of channels	128	62	32
Length of time samples	1000	1000	1000
Frequency of samples	256	250	250
Number of classes	4	11	10
Size of train dataset	400	105	400
Size of test dataset	100	27	100

**Table 3:** Accuracy (%) across different methods on Dataset I. **Bold** values indicate the best performance in each subject.

Method	S01	S02	S03	S04	S05	S06	S07	S08	S09	S10	Avg
ShallowConvNet [27,28]	33.23 ± 4.15	32.33 ± 3.46	36.00 ± 2.00	33.33 ± 3.33	32.67 ± 5.35	31.48 ± 3.93	30.67 ± 1.90	34.80 ± 6.42	29.67 ± 3.80	32.67 ± 3.65	32.69 ± 3.80
DeepConvNet [27,28]	34.45 ± 2.97	35.00 ± 6.77	36.45 ± 6.07	33.67 ± 5.19	32.67 ± 3.46	37.41 ± 4.42	36.33 ± 3.42	37.60 ± 4.56	34.00 ± 1.90	37.00 ± 5.45	35.46 ± 4.42
EEGNet [17,29]	29.67 ± 3.85	28.33 ± 4.41	32.40 ± 2.97	37.00 ± 2.17	30.33 ± 5.94	28.52 ± 3.10	29.00 ± 6.08	32.00 ± 8.37	26.33 ± 5.94	29.00 ± 4.65	30.26 ± 4.75
RACNN [30]	29.23 ± 3.03	23.00 ± 2.98	26.45 ± 2.19	26.00 ± 2.24	25.67 ± 2.53	25.19 ± 3.10	28.00 ± 1.39	22.89 ± 3.35	24.00 ± 3.46	27.67 ± 6.52	25.81 ± 3.08
EEG-ChannelNet [31]	35.67 ± 4.34	36.00 ± 2.79	32.00 ± 3.16	31.00 ± 2.24	32.00 ± 4.15	34.07 ± 1.01	31.33 ± 2.17	35.23 ± 3.35	30.67 ± 0.91	36.33 ± 4.31	33.43 ± 2.84
Conformer [22]	39.67 ± 6.07	33.67 ± 5.06	38.89 ± 7.56	36.33 ± 6.60	36.67 ± 4.08	38.52 ± 3.56	37.00 ± 6.71	40.00 ± 4.69	33.00 ± 5.70	42.67 ± 5.96	37.64 ± 5.60
LMDA-Net [32]	36.89 ± 5.93	35.00 ± 3.54	37.23 ± 7.01	29.00 ± 2.53	32.67 ± 4.50	33.33 ± 4.72	30.33 ± 2.74	32.45 ± 4.98	30.67 ± 6.73	28.67 ± 2.98	32.62 ± 4.57
AISR (SPWVD+CNN) [33]	30.08 ± 0.44	31.92 ± 2.32	33.34 ± 1.05	33.05 ± 3.81	37.72 ± 1.37	29.17 ± 3.98	28.46 ± 0.93	35.37 ± 1.96	33.59 ± 3.28	34.65 ± 2.58	32.74 ± 2.17
EEG-Deformer [21]	42.41 ± 11.47	**47.00 ± 2.74**	40.59 ± 3.22	43.00 ± 9.08	**41.00 ± 11.18**	46.27 ± 7.45	41.00 ± 3.25	42.94 ± 8.32	42.00 ± 8.37	48.75 ± 2.55	43.50 ± 6.76
D-FaST [20]	35.67 ± 2.97	34.67 ± 2.98	37.23 ± 4.15	30.33 ± 3.80	31.67 ± 4.25	31.48 ± 5.24	34.00 ± 9.17	34.89 ± 4.60	30.00 ± 2.64	40.67 ± 7.87	34.06 ± 4.77
**STCCA (Proposed)**	**45.88 ± 4.92**	46.00 ± 8.22	**45.88 ± 6.44**	**44.00 ± 5.48**	40.00 ± 3.54	**46.67 ± 6.33**	**43.00 ± 6.71**	**47.06 ± 4.16**	**46.00 ± 9.62**	**50.00 ± 9.35**	**45.45 ± 6.48**

**Table 4:** Accuracy (%) across different methods on Dataset II. **Bold** values indicate the best performance in each subject.

Method	S01	S02	S03	S04	S05	S06	Avg
ShallowConvNet [27,28]	11.98 ± 4.19	8.90 ± 3.02	13.30 ± 5.79	24.07 ± 8.83	13.52 ± 3.59	16.47 ± 11.31	14.71 ± 6.12
DeepConvNet [27,28]	14.97 ± 6.52	17.91 ± 4.03	19.34 ± 8.52	25.38 ± 4.05	26.81 ± 3.53	21.18 ± 5.26	20.93 ± 5.32
EEGNet [17,29]	10.44 ± 4.04	16.37 ± 2.87	16.59 ± 6.71	15.05 ± 5.65	25.38 ± 4.05	14.12 ± 5.26	16.33 ± 4.76
RACNN [30]	13.52 ± 3.59	19.45 ± 4.28	22.42 ± 5.13	19.45 ± 4.28	20.88 ± 6.30	15.29 ± 3.22	18.50 ± 4.47
EEG-ChannelNet [31]	10.33 ± 6.21	17.80 ± 6.37	11.76 ± 6.11	23.85 ± 6.09	32.75 ± 2.71	23.74 ± 5.42	20.04 ± 5.49
Conformer [22]	10.55 ± 4.42	16.26 ± 7.59	14.97 ± 6.52	19.56 ± 10.58	19.01 ± 12.65	20.00 ± 3.22	16.73 ± 7.50
LMDA-Net [32]	16.26 ± 5.66	13.30 ± 5.79	14.70 ± 5.63	25.60 ± 7.51	16.48 ± 6.28	18.82 ± 4.92	17.53 ± 5.97
AISR (SPWVD+CNN) [33]	12.69 ± 1.06	12.15 ± 0.91	13.71 ± 2.51	12.53 ± 0.08	13.23 ± 3.95	11.11 ± 0.60	12.57 ± 1.52
EEG-Deformer [21]	**20.00 ± 11.18**	21.00 ± 7.68	22.02 ± 5.68	25.00 ± 1.20	20.00 ± 2.92	23.56 ± 5.95	21.93 ± 5.77
D-FaST [20]	13.41 ± 6.00	15.05 ± 5.65	16.37 ± 6.15	28.24 ± 7.65	22.42 ± 7.48	20.00 ± 3.22	19.25 ± 6.03
**STCCA (Proposed)**	18.23 ± 3.57	**22.23 ± 5.04**	**23.50 ± 2.50**	**31.25 ± 5.70**	**35.50 ± 7.43**	**24.72 ± 4.82**	**25.91 ± 4.84**

**Table 5:** Accuracy (%) across different methods on Dataset III. **Bold** values indicate the best performance in each subject.

Method	S01	S02	S03	S04	S05	S06	Avg
ShallowConvNet [27,28]	18.82 ± 9.67	19.12 ± 5.63	17.86 ± 7.14	21.18 ± 9.84	21.18 ± 6.71	20.00 ± 3.22	19.69 ± 7.04
DeepConvNet [27,28]	12.94 ± 12.75	15.29 ± 5.26	18.57 ± 3.91	12.94 ± 7.67	14.12 ± 9.84	20.00 ± 7.89	15.64 ± 7.89
EEGNet [17,29]	20.59 ± 3.42	14.12 ± 3.22	22.86 ± 9.31	17.65 ± 4.80	20.00 ± 8.92	18.82 ± 10.52	19.01 ± 6.70
RACNN [30]	27.06 ± 3.22	24.71 ± 4.92	21.43 ± 2.04	24.71 ± 6.44	16.47 ± 4.92	23.53 ± 8.32	22.99 ± 4.98
EEG-ChannelNet [31]	20.00 ± 7.89	24.71 ± 6.44	14.29 ± 3.50	21.18 ± 3.22	25.88 ± 5.26	16.47 ± 4.92	20.42 ± 5.21
Conformer [22]	22.35 ± 2.63	21.18 ± 5.26	21.43 ± 7.14	25.88 ± 6.71	23.53 ± 3.06	23.53 ± 4.16	22.98 ± 4.83
LMDA-Net [32]	20.00 ± 6.71	15.29 ± 7.89	22.86 ± 13.74	22.35 ± 7.67	18.82 ± 7.67	23.53 ± 4.16	20.48 ± 7.97
AISR(SPWVD+CNN) [33]	11.88 ± 0.97	12.44 ± 1.18	11.34 ± 0.57	12.04 ± 0.67	12.10 ± 0.23	11.08 ± 1.44	11.81 ± 0.84
EEG-Deformer [21]	27.41 ± 4.16	27.06 ± 5.26	25.71 ± 3.91	28.24 ± 4.92	**30.59 ± 6.44**	23.53 ± 4.16	27.09 ± 4.81
D-FaST [20]	19.40 ± 9.09	20.00 ± 8.92	18.82 ± 2.63	18.57 ± 3.91	26.00 ± 15.17	22.35 ± 2.63	20.86 ± 7.06
**STCCA (Proposed)**	**28.24 ± 2.63**	**31.76 ± 5.26**	**28.57 ± 8.75**	**29.41 ± 4.16**	28.41 ± 5.88	**28.00 ± 5.95**	**29.07 ± 5.44**

**Table 6:** F1_score and accuracy(%) under channel dropout conditions (5% and 10%) on Dataset I.

	S01	S02	S03	S04	S05	S06	S07	S08	S09	S10
F1_score	0.4125	0.3996	0.3570	0.3852	0.4132	0.4094	0.3594	0.4452	0.3764	0.4554
Acc_5	30.00	33.33	27.00	31.67	35.00	30.56	25.83	34.00	30.83	35.83
Acc_10	32.00	29.17	27.00	32.50	33.33	30.56	26.67	34.00	30.00	33.33

Acc_5 and Acc_10 represent the accuracies when 5% and 10% of the channels are randomly dropped.

**Table 7:** Summary of parameters, FLOPs, and inference efficiency across method. **Bold** values indicate the best performance in each column.

Method	Parameters (M)	FLOPs (M)	Inference Time (ms)
EEG-ChannelNet	36.682	13,547.434	12.126
Conformer	31.155	2012.787	16.094
EEG-Deformer	**1.096**	**98.146**	6.067
D-FaST	6.386	7252.466	99.338
**STCCA (Proposed)**	7.155	2581.882	**4.597**

**Table 8:** Accuracy (%) under the LOSO cross-subject evaluation on Dataset I. **Bold** values indicate the best performance in each subject.

Method	S01	S02	S03	S04	S05	S06	S07	S08	S09	S10
ShallowConvNet	27.18	25.50	28.17	27.67	25.33	27.04	27.00	27.38	26.33	26.17
DeepConvNet	29.56	28.67	28.97	27.00	29.17	29.26	26.00	30.36	28.50	25.50
EEGNet	27.78	27.17	**30.36**	30.00	29.17	24.63	26.83	26.98	25.83	27.17
EEG-ChannelNet	29.37	26.83	27.18	26.00	27.33	27.96	**28.33**	29.37	**28.83**	**27.83**
LMDA	29.17	29.33	28.17	24.83	28.00	27.78	26.83	26.19	25.17	26.17
Deformer	28.57	25.50	29.17	26.33	27.50	26.48	26.00	27.98	25.33	25.33
D-FaST	30.00	29.00	26.98	28.83	25.83	27.22	27.00	29.54	28.67	**27.83**
**STCCA (Proposed)**	**30.36**	**29.83**	29.37	**30.17**	**29.33**	**29.30**	27.67	**30.37**	27.50	**27.83**

**Table 9:** Ablation results of LFEM and GFEM on Dataset I. **Bold** values indicate the best performance in each subject.

Method	S01	S02	S03	S04	S05	S06	S07	S08	S09	S10	Avg
w/o LFEM	40.06 ± 7.20	42.00 ± 4.47	44.71 ± 5.26	42.76 ± 7.42	38.70 ± 9.08	44.44 ± 3.93	40.05 ± 7.42	42.35 ± 9.67	42.00 ± 10.95	40.00 ± 7.07	41.71 ± 7.25
w/o GFEM	41.88 ± 9.67	43.00 ± 8.37	43.41 ± 5.26	40.36 ± 4.18	39.45 ± 4.58	43.36 ± 2.74	39.48 ± 4.92	40.59 ± 6.71	45.00 ± 3.54	43.63 ± 5.84	42.02 ± 5.58
**STCCA (Proposed)**	**45.88 ± 4.92**	**46.00 ± 8.22**	**45.88 ± 6.44**	**44.00 ± 5.48**	**40.00 ± 3.54**	**46.67 ± 6.33**	**43.00 ± 6.71**	**47.06 ± 4.16**	**46.00 ± 9.62**	**50.00 ± 9.35**	**45.45 ± 6.48**

**Table 10:** Ablation results of LFEM and GFEM on Dataset II. **Bold** values indicate the best performance in each subject.

Method	S01	S02	S03	S04	S05	S06	Avg
w/o LFEM	15.23 ± 2.62	20.50 ± 5.02	20.65 ± 3.69	15.00 ± 13.69	31.00 ± 3.69	8.00 ± 10.95	18.40 ± 4.94
w/o GFEM	17.04 ± 3.69	13.69 ± 4.13	19.89 ± 2.92	30.00 ± 20.92	30.00 ± 1.18	12.00 ± 10.92	20.44 ± 7.29
**STCCA (Proposed)**	**18.23 ± 3.57**	**22.23 ± 5.04**	**23.50 ± 2.50**	**31.25 ± 5.70**	**35.50 ± 7.43**	**24.72 ± 4.82**	**25.91 ± 4.84**

**Table 11:** Ablation results of LFEM and GFEM on Dataset III. **Bold** values indicate the best performance in each subject.

Method	S01	S02	S03	S04	S05	S06	Avg
w/o LFEM	22.35 ± 7.67	27.06 ± 6.71	22.86 ± 7.83	22.76 ± 9.84	24.71 ± 6.44	23.53 ± 8.32	23.88 ± 7.80
w/o GFEM	23.41 ± 4.16	29.41 ± 7.2	25.71 ± 6.39	20.59 ± 6.44	21.76 ± 6.71	25.49 ± 6.79	24.40 ± 6.28
**STCCA (Proposed)**	**28.24 ± 2.63**	**31.76 ± 5.26**	**28.57 ± 8.75**	**29.41 ± 4.16**	28.41 ± 5.88	**28.00 ± 5.95**	**29.07 ± 5.44**

**Table 12:** Summary of parameter counts, FLOPs, and inference efficiency across fusion strategies. **Bold** values indicate the best performance in each column.

	Parameters (M)	FLOPs (M)	Inference Time (ms)
sum	**7.155**	**2547.787**	**4.250**
cat	**7.155**	2567.283	5.473
atten	**7.155**	2548.901	4.308
CCA	**7.155**	2581.882	4.597

**Figure A1:**
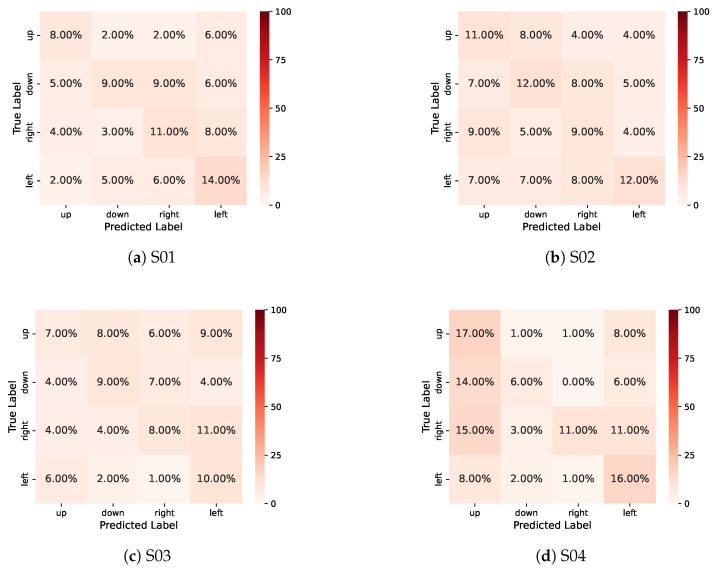

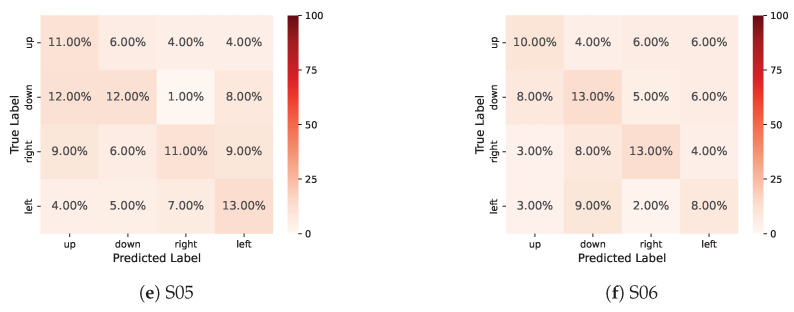
Confusion matrices of S01–S06 (**a**–**f**) on Dataset I.

**Figure A2:**
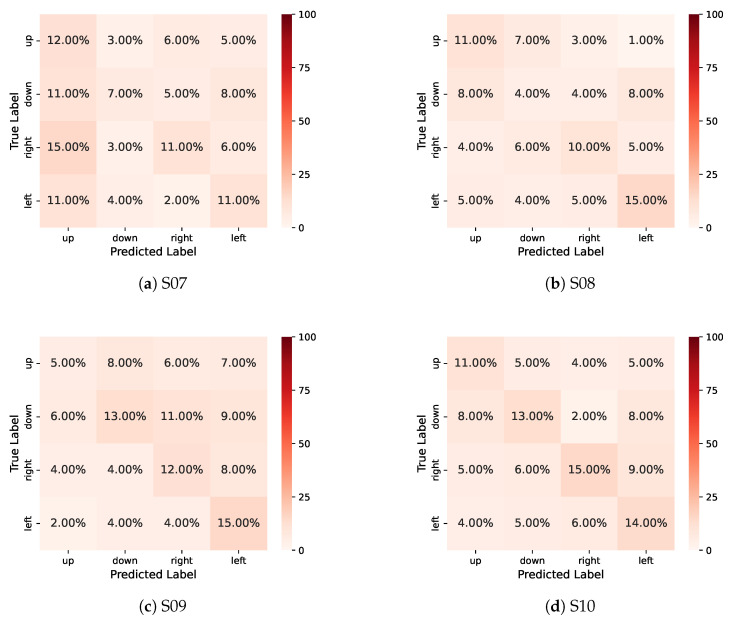
Confusion matrices of S07–S10 (**a**–**d**) on Dataset I.

## Data Availability

Our code and model are available at https://github.com/buptantEEG/STCCA (accessed on 20 October 2025). The Dataset I presented in this study are available at https://openneuro.org/datasets/ds003626/versions/2.1.2 (accessed on 4 March 2025). The Dataset II presented in this study are available at http://www.cs.toronto.edu/~complingweb/data/karaOne/karaOne.html (accessed on 14 April 2025).

## Abbreviations

The following abbreviations are used in this manuscript:

EEG	electroencephalogram
BCI	brain–computer interface
DL	deep learning
CNN	convolutional neural network
LFEM	local feature extraction module
CCA	coupled cross-attention
GFEM	global feature extraction module
Conv	convolutional layers
AG	average pooling layers
BN	batch normalization layers
LN	layer normalization
MHA	multi-head self-attention

## Appendix A

### Appendix A.1

**Table A1:** Number of trials per class per subject for Dataset I.

	S01	S02	S03	S04	S05	S06	S07	S08	S09	S10
Class0	125	150	125	150	150	135	150	125	150	150
Class1	125	150	125	150	150	135	150	125	150	150
Class2	125	150	125	150	150	135	150	125	150	150
Class3	125	150	125	150	150	135	150	125	150	150

**Table A2:** Number of trials per class per subject for Dataset II.

	S01	S02	S03	S04	S05	S06
Class0	12	12	12	12	15	12
Class1	12	12	12	12	15	12
Class2	12	12	12	12	15	12
Class3	12	12	12	12	15	12
Class4	12	12	12	12	15	12
Class5	12	12	12	12	15	12
Class6	12	12	12	12	15	12
Class7	12	12	12	12	15	12
Class8	12	12	12	12	15	12
Class9	12	12	12	12	15	12
Class10	12	12	12	12	15	12

**Table A3:** Number of trials per class per subject for Dataset III.

	S01	S02	S03	S04	S05	Sub6
Class0	50	40	50	40	50	40
Class1	50	40	50	40	50	40
Class2	50	40	50	40	50	40
Class3	50	40	50	40	50	40
Class4	50	40	50	40	50	40
Class5	50	40	50	40	50	40
Class6	50	40	50	40	50	40
Class7	50	40	50	40	50	40
Class8	50	40	50	40	50	40
Class9	50	40	50	40	50	40

## References

[1] Zhang L., Zhou Y., Gong P., Zhang D. (2024). Speech imagery decoding using EEG signals and deep learning: A survey. IEEE Trans. Cogn. Dev. Syst..

[2] Guetschel P., Ahmadi S., Tangermann M. (2024). Review of deep representation learning techniques for brain–computer interfaces. J. Neural Eng..

[3] Musso M., Hübner D., Schwarzkopf S., Bernodusson M., LeVan P., Weiller C., Tangermann M. (2022). Aphasia recovery by language training using a brain–computer interface: A proof-of-concept study. Brain Commun..

[4] Lopez-Bernal D., Balderas D., Ponce P., Molina A. (2024). Exploring inter-trial coherence for inner speech classification in EEG-based brain–computer interface. J. Neural Eng..

[5] Kamble A., Ghare P.H., Kumar V. (2023). Optimized rational dilation wavelet transform for automatic imagined speech recognition. IEEE Trans. Instrum. Meas..

[6] Rahman N., Khan D.M., Masroor K., Arshad M., Rafiq A., Fahim S.M. (2024). Advances in brain-computer interface for decoding speech imagery from EEG signals: A systematic review. Cogn. Neurodyn..

[7] Cai Z., Luo T.j., Cao X. (2024). Multi-branch spatial-temporal-spectral convolutional neural networks for multi-task motor imagery EEG classification. Biomed. Signal Process. Control.

[8] Li X., Tang J., Li X., Yang Y. (2024). CWSTR-Net: A Channel-Weighted Spatial–Temporal Residual Network based on nonsmooth nonnegative matrix factorization for fatigue detection using EEG signals. Biomed. Signal Process. Control.

[9] Nieto N., Peterson V., Rufiner H.L., Kamienkowski J.E., Spies R. (2022). Thinking out loud, an open-access EEG-based BCI dataset for inner speech recognition. Sci. Data.

[10] Kamble A., Ghare P.H., Kumar V. (2022). Deep-learning-based BCI for automatic imagined speech recognition using SPWVD. IEEE Trans. Instrum. Meas..

[11] Li C., Wang H., Liu Y., Zhu X., Song L. (2024). Silent EEG classification using cross-fusion adaptive graph convolution network for multilingual neurolinguistic signal decoding. Biomed. Signal Process. Control.

[12] Li C., Liu Y., Li J., Miao Y., Liu J., Song L. (2024). Decoding Bilingual EEG Signals With Complex Semantics Using Adaptive Graph Attention Convolutional Network. IEEE Trans. Neural Syst. Rehabil. Eng..

[13] Liu K., Yang M., Yu Z., Wang G., Wu W. (2022). FBMSNet: A filter-bank multi-scale convolutional neural network for EEG-based motor imagery decoding. IEEE Trans. Biomed. Eng..

[14] Cao L., Yu B., Dong Y., Liu T., Li J. (2024). Convolution spatial-temporal attention network for EEG emotion recognition. Physiol. Meas..

[15] Zhao W., Jiang X., Zhang B., Xiao S., Weng S. (2024). CTNet: A convolutional transformer network for EEG-based motor imagery classification. Sci. Rep..

[16] Park D., Park H., Kim S., Choo S., Lee S., Nam C.S., Jung J.Y. (2023). Spatio-temporal explanation of 3D-EEGNet for motor imagery EEG classification using permutation and saliency. IEEE Trans. Neural Syst. Rehabil. Eng..

[17] Lawhern V.J., Solon A.J., Waytowich N.R., Gordon S.M., Hung C.P., Lance B.J. (2018). EEGNet: A compact convolutional neural network for EEG-based brain–computer interfaces. J. Neural Eng..

[18] Ding Y., Robinson N., Zhang S., Zeng Q., Guan C. (2022). TSception: Capturing temporal dynamics and spatial asymmetry from EEG for emotion recognition. IEEE Trans. Affect. Comput..

[19] Chang Y., Zheng X., Chen Y., Li X., Miao Q. (2024). Spatiotemporal Gated Graph Transformer for EEG-based Emotion Recognition. IEEE Signal Process. Lett..

[20] Chen W., Wang C., Xu K., Yuan Y., Bai Y., Zhang D. (2024). D-FaST: Cognitive Signal Decoding with Disentangled Frequency-Spatial-Temporal Attention. IEEE Trans. Cogn. Dev. Syst..

[21] Ding Y., Li Y., Sun H., Liu R., Tong C., Liu C., Zhou X., Guan C. (2024). EEG-Deformer: A dense convolutional transformer for brain-computer interfaces. IEEE J. Biomed. Health Inform..

[22] Song Y., Zheng Q., Liu B., Gao X. (2022). EEG conformer: Convolutional transformer for EEG decoding and visualization. IEEE Trans. Neural Syst. Rehabil. Eng..

[23] Zhang X., Cheng X. (2024). A transformer convolutional network with the method of image segmentation for EEG-based emotion recognition. IEEE Signal Process. Lett..

[24] Vaswani A. Attention is all you need. Proceedings of the Advances in Neural Information Processing Systems 30.

[25] Zhao S., Rudzicz F. Classifying phonological categories in imagined and articulated speech. Proceedings of the 2015 IEEE International Conference on Acoustics, Speech and Signal Processing (ICASSP).

[26] Wong T.T., Yeh P.Y. (2020). Reliable Accuracy Estimates from k-Fold Cross Validation. IEEE Trans. Knowl. Data Eng..

[27] Schirrmeister R.T., Springenberg J.T., Fiederer L.D.J., Glasstetter M., Eggensperger K., Tangermann M., Hutter F., Burgard W., Ball T. (2017). Deep learning with convolutional neural networks for EEG decoding and visualization. Hum. Brain Mapp..

[28] Yang Y., Zhang X., Zhang X., Yu C. (2024). MCMTNet: Advanced network architectures for EEG-based motor imagery classification. Neurocomputing.

[29] Moctezuma L.A., Suzuki Y., Furuki J., Molinas M., Abe T. (2024). GRU-powered sleep stage classification with permutation-based EEG channel selection. Sci. Rep..

[30] Fang Z., Wang W., Ren S., Wang J., Shi W., Liang X., Fan C.C., Hou Z.G. Learning Regional Attention Convolutional Neural Network for Motion Intention Recognition Based on EEG Data. Proceedings of the Twenty-Ninth International Conference on International Joint Conferences on Artificial Intelligence.

[31] Palazzo S., Spampinato C., Kavasidis I., Giordano D., Schmidt J., Shah M. (2020). Decoding brain representations by multimodal learning of neural activity and visual features. IEEE Trans. Pattern Anal. Mach. Intell..

[32] Miao Z., Zhao M., Zhang X., Ming D. (2023). LMDA-Net: A lightweight multi-dimensional attention network for general EEG-based brain-computer interfaces and interpretability. NeuroImage.

[33] Kamble A., Ghare P.H., Kumar V., Kothari A., Keskar A.G. (2023). Spectral analysis of EEG signals for automatic imagined speech recognition. IEEE Trans. Instrum. Meas..

